# The relationship between the mode of presentation, CT‐derived body composition, systemic inflammatory grade and survival in colon cancer

**DOI:** 10.1002/jcsm.13097

**Published:** 2022-10-11

**Authors:** Allan M. Golder, Ling Kwan Ernest Sin, Fatima Alani, Ala Alasadi, Ross Dolan, David Mansouri, Paul G. Horgan, Donald C. McMillan, Campbell S. Roxburgh

**Affiliations:** ^1^ Academic Unit of Surgery University of Glasgow Glasgow UK

**Keywords:** Colon, Body composition, CT, Cancer, Sarcopenia, Inflammation

## Abstract

**Background:**

Within colorectal cancer, the systemic inflammatory response (SIR) and CT‐derived body composition, particularly the loss of lean muscle mass, are independently associated with oncological outcomes; however, no study has included both non‐metastatic and metastatic disease. The present study analyses the association between body composition, mode of presentation, SIR and survival in patients with TNM I–IV colon cancer.

**Methods:**

Patients diagnosed with colon cancer from 2011 to 2014 were identified. The SIR was stratified using systemic inflammatory grade (SIG). Staging CT scans were used to define body composition: subcutaneous fat index (SFI), visceral fat area (VFA), skeletal muscle index (SMI) and skeletal muscle density (SMD). The effect of SIG and body composition on mode of presentation and 3‐year overall survival (3‐yr OS) was analysed.

**Results:**

One thousand one hundred forty‐six patients were identified; 14%/38%/40%/8% had TNM Stage I/II/III/IV colon cancer, respectively. Patients were predominantly aged 65 + (63%), male (52%) and BMI > 25 (62%). 79%74% had a high SFI/VFA, and 56%/62% had a low SMI/SMD, respectively. Abnormal body composition was prevalent across all disease stages and associated with TNM stage—high SFI in 87%/76%/81%/68% (*P* < 0.001), high VFA in 79%/73%/75%/67% (*P* = 0.189), low SMI in 43%/60%/55%/68% (*P* < 0.001) and low SMD in 55%/65%/61%/67% (*P* = 0.094) of TNM I/II/III/IV disease, respectively. Body composition was associated with SIG—high SFI in 83%/80%/77%/78%/66% (*P* = 0.004), high VFA in 78%/78%/70%/63%/61% (*P* = 0.002), low SMI in 48%/52%/62%/62%/79% (*P* < 0.001) and low SMD in 56%/60%/62%/70%/76% (*P* < 0.001) of patients with SIG 0/1/2/3/4, respectively. After adjustment for other factors, increased SIG (OR 1.95), visceral obesity (OR 0.65) and low SMI (OR 1.61) were associated with emergency presentation. In TNM Stage II colon cancer, low SMI and low SMD were associated with worse 3‐yr OS (92% vs 87%, *P* < 0.001 and 96% vs 85%, *P* < 0.001, respectively). In TNM Stage III, a trend was seen between low SMI and SMD and 3‐yr OS (77% vs 73%, *P* = 0.091 and 76% vs 75%, *P* = 0.034, respectively). In TNM Stage IV disease, low SMI was associated with 3‐yr OS (43% vs 16%, *P* < 0.001). A trend, albeit not of significance, was seen between low SMD and 3‐yr OS (32% vs 21%, *P* = 0.366).

**Conclusions:**

The present results show that abnormal body composition is prevalent across TNM I–IV colon cancer and associated with TNM stage and SIG. Body composition is independently associated with emergency presentation and long‐term survival. Further research is required to analyse whether interventions including structured exercise programmes or attenuation of the SIR have an effect on CT‐derived body composition and oncological outcomes.

## Introduction

Cachexia is closely associated with a number of systemic illnesses including cancer and organ failure. The syndrome of cachexia, previously described as ‘an ongoing loss of skeletal muscle with or without loss of fat mass not entirely reversible with nutritional support’,^1^ is, in a similar way to the hallmarks of cancer, now recognized to be inextricably linked to inflammatory responses, in particular the systemic inflammatory response (SIR).^2^ This has resulted in cachexia being redefined as ‘weight loss, reduced BMI and reduced muscle mass and function in combination with an underlying disease that displays biochemical indices of ongoing elevated inflammatory activity’ or more simply put as ‘disease related malnutrition with inflammation’.^3^ In the context of cancer, the prevalence of cachexia varies with mode of presentation, tumour site and stage however is recognized to be particularly common in gastrointestinal and lung cancer^4^ and strongly associated with poor outcomes.

An increasing body of literature reports the association between an increased pre‐operative SIR and poor oncological outcomes following curative surgery for a number of cancer types including colorectal cancer.^5^ This SIR has been previously quantified using either hepatic acute phase proteins [predominantly modified Glasgow Prognostic Score (mGPS^6^, ^7^)] or immune haematological markers [predominantly neutrophil–lymphocyte ratio (NLR^8^)]. More recently, the synergistic effect of both of these has been reported and incorporated into an overall systemic inflammatory grade (SIG).^9^

Traditional markers of cachexia and malnutrition such as absolute BMI, weight loss and a characteristic ‘cachectic appearance’, although readily available, are now becoming less clear in the increasingly obese Western population. BMI, while readily calculated, carries the major limitation of being unable to differentiate between fat and muscle mass. This has resulted in the development of alternative anthropometric measurements,^10^ in particular image‐based body composition.

Within oncology, CT‐derived body composition is the most widely utilized anthropometric measurement due to the routine availability of CT imaging primarily obtained for cancer staging. Typically, a single CT slice is analysed for fat and muscle characteristics at the level of the third lumbar vertebrae including muscle area/density. Image analysis software (e.g. ImageJ^11^) can differentiate between muscle and fat using previously validated Hounsfield Unit (HU) thresholds (fat −190 HU to −30 HU, muscle −29 HU to +150 HU). These measurements can be subsequently stratified into normal/abnormal using a number of variables including sex, height and BMI to obtain four main measurements of body composition: subcutaneous fat index (SFI), visceral fat area (VFA), skeletal muscle index (SMI) and skeletal muscle density (SMD).

As reported in a recent systematic review,^2^ body composition and the SIR are inextricably linked, independent of tumour stage; however, the causal nature of this relationship is not clear. In particular, this relationship has been examined in either primary operable cancer or advanced cancer but not both in the same tumour type. The aim of the present study was to examine the relationship between mode of presentation, body composition, SIG and survival in a cohort of patients with TNM Stage I–IV colon cancer.

## Methods

Within Scotland, basic clinicopathological data for all new cases of colorectal cancer are prospectively collected and held within regional Managed Clinical Network (MCN) datasets. The West of Scotland Cancer Network (WoSCAN) dataset incorporates four health boards (Ayrshire and Arran, Fort Valley, Lanarkshire and Greater Glasgow and Clyde) and includes approximately half of the population of Scotland. These patients receive treatment in line with national guidelines and are followed up for a period of 3–5 years.

Patients diagnosed with colon cancer between January 2011 and December 2014 were identified from the West of Scotland MCN database. Patients undergoing curative surgery for either an elective or emergency diagnosis of TNM I–III colon cancer and all patients diagnosed with TNM IV colon cancer were included. Rectal (including rectosigmoid) tumours and those without an available BMI and/or the laboratory data required to calculate SIG^9^ (pre‐operative neutrophils/lymphocytes/C‐reactive protein/albumin) were excluded. For TNM Stage I–III patients, those patients with macroscopically involved margins (R2 resections) and those patients undergoing local/palliative procedures were excluded. Survival was updated through data linkage to the National Records of Scotland (NRS) deaths data until the end of 2018.

Socio‐economic deprivation has been stratified using the Scottish Index of Multiple Deprivation (SIMD).^12^ Tumours were staged using the TNM classification system. The pre‐operative SIR has been stratified using SIG.^9^ For TNM I–III patients, pre‐operative blood results were regarded as the most recent set of pre‐operative results in the case of elective patients within 1 month prior to surgery and in the case of emergency patients from admission to hospital. For TNM IV patients, blood results were within 1 month of diagnosis and pre‐operatively in the case of those patients undergoing surgery. Overall survival was calculated from the date of diagnosis until date of death of any cause. All patients were followed up for a minimum of 4 years following diagnosis.

### Anthropometric measurements

A single CT image was obtained at the level of the third lumbar vertebra from the routine staging scan carried out at time of cancer diagnosis. Scans with significant movement artefact or a missing region of interest were excluded. Images were analysed using a freeware program—NIH ImageJ Version 1.52 (National Institutes of Health, USA).

Standard HU ranges were used to define adipose tissue (−190 to −30) and skeletal muscle (−29 to +150). Measurements were made of total subcutaneous fat area, visceral fat area, skeletal muscle area (all cm^2^) and skeletal muscle density (mean HU). Subcutaneous fat area and skeletal muscle area were subsequently normalized for height^2^ to create subcutaneous fat and skeletal muscle indices (SFI and SMI, respectively, cm^2^/m^2^). Stratification of anthropometric measurements as normal/abnormal was carried out for SFI, VFA, SMI and SMD as previously described by Ebadi,^13^ Doyle,^14^ Martin^15^ and Xiao,^16^ respectively (*Table* 1).

**Table 1:** Classification of abnormal CT‐derived body composition

High subcutaneous fat index		
Males	Ebadi^13^	>50 cm^2^ m^2^
Females		>42 cm^2^ m^2^

High visceral fat area		
Males	Doyle^14^	VFA > 160
Females		VFA > 80

Low skeletal muscle index		
Males, BMI < =25	Martin^15^	SMI < 45
Males, MBI > 25		SMI < 53
Females, BMI < =25		SMI < 39
Females, BMI > 25		SMI < 41

Low skeletal muscle density		
Males	Xiao^16^	<35.5 HU
Females		<32.5 HU

Ethical approval was granted for this project from the Public Benefit and Privacy Panel (PBPP) for Health and Social Care (NHS Scotland) and Caldicott Guardian Approval.

### Statistical analysis

The relationship between TNM stage, SIG and CT‐derived body composition has been carried out using the chi‐squared test. Results are displayed as the total number of patients within each TNM stage/SIG category and percentage of the total number with the relevant abnormal CT‐derived body composition marker.

The association between clinicopathological factors including CT‐derived body composition has been carried out using binary logistic regression to calculate odds ratios (ORs) and 95% confidence intervals (95% CI). Variables with a *P*‐value of <0.1 on univariate analysis were entered into the multivariate model.

Three‐year overall survival was calculated using a life table approach, and results were displayed as percentage 3‐year survival and percentage standard error. Where there were fewer than 10 patients in a group, survival analysis was not carried out due to potential inaccuracies resulting from small sample size. On survival analysis, statistical significance was calculated using the log‐rank test.

## Results

### Patient characteristics

Patient characteristics are shown in *Table* 2. The majority of patients included within the present study presented electively (79%) with TNM II/III disease (38%/40%). 79%/74% had a high subcutaneous fat index/visceral fat area, respectively. 56%/62% of patients had a low skeletal muscle index/skeletal muscle density, respectively. Of 1107 patients who underwent surgery, there were 14 post‐operative deaths (1%). There were 395 deaths during the follow‐up period for whom the median time from diagnosis to death was 26 months. All patients were followed up for a minimum of 4 years from date of diagnosis.

**Table 2:** Patient demographics

Variable	Total
Host factors	
Age	1146
<65	422 (37%)
65–74	387 (34%)
75+	337 (29%)
Sex	1146
Male	590 (52%)
Female	556 (48%)
SIMD	1146
1	344 (30%)
2	238 (21%)
3	179 (16%)
4	178 (16%)
5	207 (18%)
Smoking	1123
Non‐smoker	542 (48%)
Ex‐smoker	408 (36%)
Smoker	173 (15%)
BMI	1146
<18.5	30 (3%)
18.5–24.9	409 (36%)
25–29.9	389 (34%)
30–34.9	203 (18%)
35+	115 (10%)
ASA	1067
1	92 (9%)
2	595 (56%)
3	348 (33%)
4	32 (3%)
Charlson score	1047
0	661 (63%)
1	266 (26%)
2	97 (9%)
3+	23 (2%)
Pre‐op anaemia	1146
None	630 (55%)
Mild	298 (26%)
Severe	218 (19%)
SIG	1146
0	404 (35%)
1	268 (23%)
2	213 (19%)
3	151 (13%)
4	110 (10%)
Tumour factors	
TNM stage	1146
I	159 (14%)
II	431 (38%)
III	463 (40%)
IV	93 (8%)
Site	1136
Right	599 (53%)
Left	537 (47%)
Differentiation	1122
Mod/well	914 (82%)
Poor	208 (19%)
EMVI	1083
Negative	565 (52%)
Positive	518 (48%)
CT‐derived body composition	
Subcutaneous fat index	1146
Normal	242 (21%)
High	904 (79%)
Visceral fat area	1146
Normal	295 (26%)
High	851 (74%)
Skeletal muscle index	1146
Normal	500 (44%)
Low	646 (56%)
Skeletal muscle density	1146
Normal	436 (38%)
Low	710 (62%)

### Association between TNM stage, SIG and anthropometric measurements

#### BMI‐defined obesity

**Figure 1:**
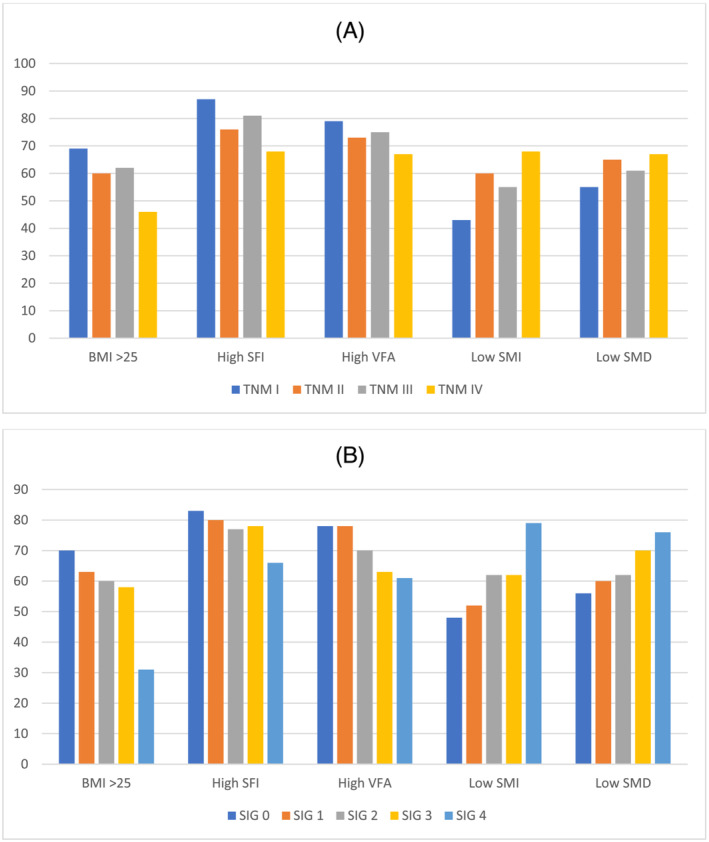
The relationship between CT‐derived body composition3 measures (%) and TNM stage (A) and systemic inflammatory grade (B).

As shown in *Table* 3A, within the overall cohort, there was a significant association between TNM stage and proportion of patients with a high BMI (>25)—69%/60%/62%/46% (*p* = 0.042) of patients with TNM Stage I/II/III/IV disease, respectively (*Figure* 1A). When patients were subgrouped by SIG, no significant association was seen between TNM stage and BMI defined obesity within any subgroup.

**Table 3:** Association between markers of body composition, SIG and TNM stage (TNM I‐IV colon cancer)

3a BMI > 25												
		TNM stage										*P*
		1		2		3		4		Total		
		*n*	%	*n*	%	*n*	%	*n*	%	n	%	
SIG	0	97	71%	139	68%	157	72%	11	55%	404	70%	0.618
	1	36	72%	102	60%	113	67%	17	41%	268	63%	0.106
	2	20	55%	90	58%	85	61%	18	67%	213	60%	0.859
	3	5	‐	57	65%	63	54%	26	50%	151	58%	0.529
	4	1	‐	43	33%	45	31%	21	24%	110	31%	0.426
	Total	159	69%	431	60%	463	62%	93	46%	1146	61%	0.004
	*P*	0.582		0.001		<0.001		0.095		<0.001		

3b High subcutaneous fat index												
		TNM stage										*P*
		I		II		III		IV		Total		
		*n*	%	*n*	%	*n*	%	*n*	%	*n*	%	
SIG	0	97	87%	139	81%	157	84%	11	64%	404	83%	0.212
	1	36	92%	102	76%	113	82%	17	71%	268	80%	0.126
	2	20	80%	90	73%	85	84%	18	61%	213	77%	0.145
	3	5	‐	57	81%	63	76%	26	69%	151	78%	0.413
	4	1	‐	43	58%	45	71%	21	71%	110	66%	0.474
	Total	159	87%	431	76%	463	81%	93	68%	1146	79%	<0.001
	*P*	0.642		0.040		0.260		0.955		0.004		

3c High visceral fat area												
		TNM stage										*P*
		I		II		III		IV		Total		
		*n*	%	*n*	%	*n*	%	*N*	%	*n*	%	
SIG	0	97	80%	139	81%	157	77%	11	46%	404	78%	0.051
	1	36	83%	102	76%	113	79%	17	71%	268	78%	0.680
	2	20	60%	90	69%	85	74%	18	72%	213	70%	0.630
	3	5	‐	57	68%	63	76%	26	73%	151	63%	0.890
	4	1	‐	43	61%	45	60%	21	62%	110	61%	0.880
	Total	159	79%	431	73%	463	75%	93	67%	1146	74%	0.189
	*P*	0.281		0.056		0.152		0.511		0.002		

3d Low skeletal muscle index												
		TNM stage										P
		1		2		3		4		Total		
		*n*	%	*n*	%	*n*	%	*n*	%	*n*	%	
SIG	0	97	41%	139	48%	157	51%	11	55%	404	48%	0.476
	1	36	44%	102	60%	113	48%	17	53%	268	52%	0.249
	2	20	60%	90	64%	85	58%	18	78%	213	62%	0.416
	3	5	‐	57	70%	63	59%	26	65%	151	62%	0.017
	4	1	‐	43	77%	45	80%	21	81%	110	79%	0.925
	Total	159	43%	431	60%	463	55%	93	68%	1146	56%	<0.001
	*P*	0.108		0.002		0.004		0.272		<0.001		

3e Low skeletal muscle density												
		TNM stage										P
		1		2		3		4		Total		
		*n*	%	*n*	%	*n*	%	*n*	%	*n*	%	
SIG	0	97	50%	139	62%	157	55%	11	64%	404	56%	0.265
	1	36	58%	102	64%	113	59%	17	47%	268	60%	0.606
	2	20	70%	90	62%	85	61%	18	61%	213	62%	0.905
	3	5	‐	57	68%	63	70%	26	73%	151	70%	0.939
	4	1	‐	43	79%	45	71%	21	81%	110	76%	0.702
	Total	159	55%	431	65%	463	61%	93	67%	1146	62%	0.094
	*P*	0.402		0.286		0.155		0.227		<0.001		

In the overall cohort, there was a significant association between SIG and proportion of patients with a high BMI—70%/63%/60%/58%/31% (*P* < 0.001) of patients with SIG 0/1/2/3/4, respectively (*Figure* 1B). When patients were subgrouped by TNM stage, a significant association was seen in the TNM Stage II cohort—68%/60%/58%/65%/33% (*P* = 0.001)—and TNM III cohort—72%/67%/61%/54%/31% (*P* < 0.001) of patients for SIG 0/1/2/3/4, respectively.

#### CT‐derived body composition: Subcutaneous fat index

As shown in *Table* 3B, within the overall cohort, there was a significant association between TNM stage and the proportion of patients with a high SFI—87%/76%/81%/68% (*P* < 0.001) of patients with TNM I/II/III/IV disease, respectively (*Figure* 1A). When patients were subgrouped by SIG, no significant association was seen between TNM stage and SFI within any subgroup.

In the overall cohort, there was a significant association between SIG and the proportion of patients with a high SFI—83%/80%/77%/78%/66% (*P* = 0.004) of patients with SIG 0/1/2/3/4, respectively (*Figure* 1B). When patients were subgrouped by TNM stage, a significant association was seen in the TNM Stage II cohort—81%/76%/73%/81%/58% (*P* = 0.040) for SIG 0/1/2/3/4, respectively.

#### CT‐derived body composition: Visceral fat area

As shown in *Table* 3C, within the overall cohort, there was no significant association (*P* = 0.189) between TNM stage and high VFA (*Figure* 1A). When patients were subgrouped by SIG, a borderline significant association was seen in the SIG 0 cohort—80%/81%/77%/46% (*P* = 0.051) of patients for TNM I/II/III/IV disease, respectively.

In the overall cohort, there was a significant association between SIG and proportion of patients with high VFA—78%/78%/70%/63%/61% (*P* = 0.002) of patients for SIG 0/1/2/3/4, respectively (*Figure* 1B). When patients were subgrouped by TNM stage, a borderline significant association was seen in the TNM Stage II cohort—81%/76%/69%/68%/61% (*P* = 0.056) of patients for SIG 0/1/2/3/4, respectively.

#### CT‐derived body composition: Skeletal muscle index

As shown in *Table* 3D, within the overall cohort, there was a significant association between TNM stage and proportion of patients with a low SMI—43%/60%/55%/68% (*P* < 0.001) of patients with TNM Stage I/II/III/IV disease, respectively (*Figure* 1A). When patients were subgrouped by SIG, a significant association was seen in the SIG 3 cohort—70%/59%/65% (*P* = 0.017) of patients with TNM Stage II/III/IV disease, respectively (*n* < 10 for TNM Stage I).

In the overall cohort, there was a significant association between SIG and proportion of patients with a low SMI—48%/52%/62%/62%/79% (*P* < 0.001) of patients with a SIG 0/1/2/3/4, respectively (*Figure* 1B). When patients were subgrouped by TNM stage, a significant association was seen in the TNM Stage II cohort—48%/60%/64%/70%/77% (*P* = 0.002) for SIG 0/1/2/3/4, respectively, and in the TNM Stage III cohort—51%/48%/58%/59%/80% (*P* = 0.004) for SIG 0/1/2/3/4, respectively.

#### CT‐derived body composition: Skeletal muscle density

As shown in *Table* 3E, within the overall cohort, there was no significant association between TNM stage and the proportion of patients with low SMD—55%/65%/61%/67% (*P* = 0.094) with TNM I/II/III/IV disease, respectively (*Figure* 1A). When patients were subgrouped by SIG, no significant association was seen between TNM stage and SMD within any subgroup.

In the overall cohort, there was a significant association between SIG and proportion of patients with low SMD—56%/60%/62%/70%/76% (*P* < 0.001) with SIG 0/1/2/3/4, respectively (*Figure* 1B). When patients were subgrouped by TNM stage, no significant association was seen between SIG and SMD within any subgroup.

### Association between body composition and mode of presentation (*Table* 4)

**Table 4:** Association between clinicopathological factors and mode of presentation

Variable	UVA		MVA	
	OR (95% CI)	*P*	OR (95% CI)	*P*
Age	0.87 (0.73–1.04)	0.118	‐	‐
Sex	1.31 (0.98–1.73)	0.066	1.44 (1.00–2.08)	0.049
SIMD	0.77 (0.90–1.08)	0.765	‐	‐
Smoking	1.35 (1.11–1.63)	0.002	1.34 (1.05–1.72)	0.019
BMI	0.66 (0.56–0.77)	<0.001	‐	0.821
ASA	1.40 (1.11–1.75)	0.004	‐	0.053
Charlson score	0.62 (0.49–0.79)	<0.001	0.47 (0.35–0.64)	<0.001
Pre‐op anaemia	1.08 (0.90–1.29)	0.412	‐	‐
SIG	2.07 (1.84–2.32)	<0.001	1.95 (1.70–2.25)	<0.001
TNM stage	1.76 (1.47–2.12)	<0.001	‐	0.097
Site	1.00 (0.75–1.33)	0.988	‐	‐
Differentiation	1.07 (0.74–1.54)	0.719	‐	‐
EMVI	2.80 (2.05–3.83)	<0.001	2.04 (1.38–3.01)	<0.001
High SFI	0.50 (0.36–0.69)	<0.001	‐	0.214
High VFA	0.47 (0.35–0.63)	<0.001	0.65 (0.43–0.96)	0.032
Low SMI	2.28 (1.68–3.10)	<0.001	1.61 (1.09–2.37)	0.016
Low SMD	1.29 (0.96–1.73)	0.095	‐	0.509

The relationship between mode of presentation and clinicopathological factors including body composition factors within TNM I–III disease is shown in *Table* 4. On univariate analysis, sex (*P* = 0.066), smoking (*P* = 0.002), BMI (*P* < 0.001), ASA (*P* = 0.004), Charlson score (*P* < 0.001), SIG (*P* < 0.001), TNM stage (*P* < 0.001), EMVI (*P* < 0.001), SFI (*P* < 0.001), VFA (*P* < 0.001), SMI (*P* < 0.001) and SMD (*P* = 0.095) were associated with mode of presentation. When those factors significant on univariate analysis were entered into the multivariate model, sex (OR 1.44, *P* = 0.049), smoking (OR 1.34, *P* = 0.019), Charlson score (OR 0.47, *P* < 0.001), SIG (OR 1.95, *P* < 0.001), EMVI (OR 2.04, *P* < 0.001), VFA (OR 0.65, *P* = 0.032) and SMI (OR 1.61, *P* = 0.016) remained significant for emergency presentation.

### Association between body composition, SIG and 3‐year survival in TNM Stage II colon cancer

The relationship between body composition, SIG and 3‐year overall survival in TNM Stage II colon cancer is shown in *Table* 5. Three‐year OS within the whole cohort was 89%. When stratified by SIG, overall survival ranged from 95% (SIG 0) to 73% (SIG 4).

**Table 5:** Three‐year overall survival stratified by SIG and body composition in TNM Stage II colon cancer

Subcutaneous fat index								
		Normal		High		Total		*P*
SIG		*n*	% 3 year OS (%SE)	*n*	% 3 year OS (%SE)	*n*	% 3 year OS (%SE)	
	0	27	93% (SE 5%)	111	95% (SE 2%)	138	95% (SE 2%)	0.197
	1	25	92% (SE 5%)	77	90% (SE 3%)	102	90% (SE 3%)	0.887
	2	24	88% (SE 7%)	66	89% (SE 4%)	90	89% (SE 3%)	0.851
	3	10	60% (SE 15%)	46	89% (SE 5%)	56	84% (SE 5%)	0.001
	4	16	94% (SE 6%)	25	60% (SE 10%)	41	73% (SE 7%)	0.040
	Total	102	88% (SE 3%)	325	89% (SE 2%)	427	89% (SE 2%)	0.254
	*P*	0.003		<0.001		<0.001		

Visceral fat area								
		Normal		High		Total		*P*
SIG	0	26	96% (SE 4%)	112	95% (SE 2%)	138	95% (SE 2%)	0.735
	1	25	100%	77	87% (SE 4%)	102	90% (SE 3%)	0.661
	2	28	86% (SE 7%)	62	90% (SE 4%)	90	89% (SE 3%)	0.971
	3	18	83% (SE 9%)	38	84% (SE 6%)	56	84% (SE 5%)	0.082
	4	16	81% (SE 10%)	25	68% (SE 9%)	41	73% (SE 7%)	0.455
	Total	113	90% (SE 3%)	314	89% (SE 2%)	427	89% (SE 2%)	0.482
	P	0.011		<0.001		<0.001		

Skeletal muscle index								
		Normal		Low		Total		*P*
SIG	0	72	96% (SE 2%)	66	94% (SE 3%)	138	95% (SE 2%)	0.237
	1	41	88% (SE 5%)	61	92% (SE 4%)	102	90% (SE 3%)	0.838
	2	32	94% (SE 4%)	58	86% (SE 5%)	90	89% (SE 3%)	0.008
	3	17	88% (SE 8%)	39	82% (SE 6%)	56	84% (SE 5%)	0.649
	4	10	90% (SE 9%)	31	68% (SE 8%)	41	73% (SE 7%)	0.220
	Total	172	92% (SE 2%)	255	87% (SE 2%)	427	89% (SE 2%)	<0.001
	*P*	0.135		0.002		<0.001		

Skeletal muscle density								
		Normal		Low		Total		*P*
SIG	0	53	100%	85	92% (SE 3%)	138	95% (SE 2%)	0.035
	1	37	95% (SE 4%)	65	88% (SE 4%)	102	90% (SE 3%)	0.041
	2	34	94% (SE 4%)	56	86% (SE 5%)	90	89% (SE 3%)	0.017
	3	18	89% (SE 7%)	38	82% (SE 6%)	56	84% (SE 5%)	0.225
	4	9		32	66% (SE 8%)	41	73% (SE 7%)	0.013
	Total	151	96% (SE 2%)	276	85% (SE 2%)	427	89% (SE 2%)	<0.001
	*P*	0.502		<0.001		<0.001		

#### SFI

Within the overall cohort of patients, SFI did not have a significant effect on survival (3‐yr OS 88% vs 89%, *P* = 0.254); however, there was a significant association between SFI and 3‐yr OS in the SIG 3 (60% vs 89%, *P* = 0.001) and SIG 4 (94% vs 60%, *P* = 0.040) subgroups. SIG was associated with worse survival independent of SFI in both the normal SFI and high SFI subgroups—3‐yr OS 93%/92%/88%/60%/94% (*P* = 0.003) and 95%/90%/89%/89%/60% (*P* < 0.001) for SIG 0/1/2/3/4, respectively.

VFA

Within the overall cohort of patients, VFA did not have a significant effect on survival (3‐yr OS 90% vs 89%, *P* = 0.482). When patients were subgrouped by SIG, no significant effect on of VFA on 3‐yr OS was seen in any SIG subgroup. SIG was associated with worse survival independent of VFA in both the normal VFA and high VFA subgroups—3‐yr OS 96%/100%/86%/83%/81% (*P* = 0.011) and 95%/87%/90%/84%/68% (*P* < 0.001) for SIG 0/1/2/3/4, respectively.

#### SMI

Within the overall cohort of patients, low SMI was associated with survival (3‐yr OS 92% vs 87%, *P* = 0.001). When patients were subgrouped by SIG, an independent effect of SMI on survival was only seen in the SIG 2 subgroup (3‐yr OS 94% vs 86%, *P* = 0.008). SIG was associated with survival only in the low SMI group—3‐yr OS 94%/92%/86%/82%/68% (*P* = 0.001) for SIG 0/1/2/3/4, respectively.

#### SMD

Within the overall cohort of patients, low SMD was associated with poorer survival (3‐yr OS 96% vs 85%, *P* < 0.001). When patients were subgrouped by SIG, an independent effect of SMD on survival was seen in the SIG 0 (100% vs 92%, *P* = 0.035), SIG 1 (95% vs 88%, *P* = 0.041), SIG 2 (94% vs 86%, *P* = 0.017) and SIG 4 (*P* = 0.013) subgroups. SIG was associated with survival only in the low SMD group—3‐yr OS 92%/88%/86%/82%/66% (*P* < 0.001) for SIG 0/1/2/3/4, respectively.

### Association between body composition, SIG and 3‐year overall survival in TNM Stage III colon cancer

The relationship between body composition, SIG and 3‐year overall survival in TNM Stage III colon cancer is shown in *Table* 6. Three‐year OS within the whole cohort was 75%. When stratified by SIG, overall survival ranged from 83% (SIG 0) to 56% (SIG 4).

**Table 6:** Three‐year overall survival stratified by SIG and body composition in TNM Stage III colon cancer

Subcutaneous fat index								
		Normal		High		Total		*P*
SIG	0	25	76% (SE 9%)	129	84% (SE 3%)	154	83% (SE 3%)	0.328
	1	19	79% (SE 9%)	92	79% (SE 4%)	111	79% (SE 4%)	0.771
	2	14	50% (SE 13%)	69	78% (SE 5%)	83	73% (SE 5%)	0.167
	3	15	67% (SE 12%)	47	64% (SE 7%)	62	65% (SE 6%)	0.757
	4	13	38% (SE 13%)	30	63% (SE 9%)	43	56% (SE 8%)	0.022
	Total	86	65% (SE 5%)	367	78% (SE 2%)	453	75% (SE 2%)	0.029
	*P*	0.006		0.023		<0.001		

Visceral fat area								
		Normal		High		Total		*P*
SIG	0	36	81% (SE 7%)	118	84% (SE 3%)	154	83% (SE 3%)	0.559
	1	24	71% (SE 9%)	87	82% (SE 4%)	111	79% (SE 4%)	0.532
	2	21	52% (SE 11%)	62	81% (SE 5%)	83	73% (SE 5%)	0.026
	3	15	67% (SE 12%)	47	64% (SE 7%)	62	65% (SE 6%)	0.934
	4	18	50% (SE 12%)	25	60% (SE 10%)	43	56% (SE 8%)	0.390
	Total	114	67% (SE 4%)	339	78% (SE 2%)	453	75% (SE 2%)	0.024
	*P*	0.055		0.014		<0.001		

Skeletal muscle index								
		Normal		Low		Total		*P*
SIG	0	75	85% (SE 4%)	79	81% (SE 4%)	154	83% (SE 3%)	0.610
	1	59	85% (SE 5%)	52	73% (SE 6%)	111	79% (SE 4%)	0.027
	2	36	69% (SE 8%)	47	77% (SE 6%)	83	73% (SE 5%)	0.436
	3	26	54% (SE 10%)	36	72% (SE 7%)	62	65% (SE 6%)	0.377
	4	8	‐	35	54% (SE 8%)	43	56% (SE 8%)	0.266
	Total	204	77% (SE 3%)	249	73% (SE 3%)	453	75% (SE 2%)	0.091
	*P*	0.004		0.013		<0.001		

Skeletal muscle density								
		Normal		Low		Total		*P*
SIG	0	71	85% (SE 4%)	83	82% (SE 4%)	154	83% (SE 3%)	0.178
	1	46	83% (SE 6%)	65	77% (SE 5%)	111	79% (SE 4%)	0.586
	2	33	67% (SE 8%)	50	78% (SE 6%)	83	73% (SE 5%)	0.567
	3	19	63% (SE 11%)	43	65% (SE 7%)	62	65% (SE 6%)	0.660
	4	13	54% (SE 14%)	30	57% (SE 9%)	43	56% (SE 8%)	0.805
	Total	182	76% (SE 3%)	271	75% (SE 3%)	453	75% (SE 2%)	0.034
	*P*	0.063		0.026		<0.001		

#### SFI

Within the overall cohort of patients, low SFI was associated with poorer survival (3‐yr OS 65% vs 78%, *P* = 0.0.029). When patients were subgrouped by SIG an independent effect of SFI on survival was only seen in the SIG 4 subgroup (38% vs 63%, *P* = 0.022). SIG was associated with worse survival independent of SFI in both the normal SFI and high SFI subgroups—3‐yr OS 76%/79%/50%/67%/38% (*P* = 0.006) and 84%/79%/78%/64%/63% (*P* = 0.023) for SIG 0/1/2/3/4, respectively.

#### VFA

Within the overall cohort of patients, low VFA was associated with poorer survival (3‐yr OS 67% vs 78%, *P* = 0.024). When patients were subgrouped by SIG, an independent effect of VFA on survival was only seen in the SIG 2 subgroup (52% vs 81%, *P* = 0.026). SIG was associated with worse survival independent of VFA in the high VFA subgroup—3‐yr OS 84%/82%/81%/64%/60% (*P* = 0.026). Although not reaching statistical significant a trend was seen in the low VFA subgroup—3‐yr OS 81%/71%/52%/67%/50% (*P* = 0.055).

#### SMI

Within the overall cohort of patients, SMI did not have a significant effect on survival (3‐yr OS 77% vs 73%, *P* = 0.091). When patients were subgrouped by SIG, an independent effect of SMI on survival was only seen in the SIG 1 cohort (85% vs 73%,*Pp* = 0.027). SIG was associated with worse survival independent of SMI in both the normal SMI and low SMI subgroups—3‐yr OS 85%/85%/69%/54% (*P* = 0.007) and 81%/73%/77%/72%/54% (*P* = 0.013) for SIG 0/1/2/3/4, respectively.

#### SMD

Within the overall cohort of patients, low SMD was associated with worse survival (3‐yr OS 76% vs 75%, *P* = 0.034). When patients were subgrouped by SIG, no significant effect on of SMD on 3‐yr OS was seen in any SIG subgroup SIG was associated with worse survival independent of SMD only in the low SMD subgroup—3‐yr OS 82%/77%/78%/65%/57% (*P* = 0.026).

### Association between body composition, SIG and 3‐year overall survival in TNM Stage IV colon cancer

The relationship between body composition, SIG and 3‐year overall survival in TNM Stage IV colon cancer is shown in *Table* 7. Three‐year OS within the whole cohort was 35%. When stratified by SIG, overall survival ranged from 64% (SIG 0) to 10% (SIG 4).

**Table 7:** Three‐year overall survival stratified by SIG and body composition in TNM Stage IV colon cancer

Subcutaneous fat index								
		Normal		High		Total		*P*
SIG	0	4	‐	7	‐	11	64% (SE 15%)	0.071
	1	5	‐	12	50% (SE 14%)	17	41% (SE 12%)	0.011
	2	7	‐	11	27% (SE 13%)	18	22% (SE 10%)	0.405
	3	8	‐	18	17% (SE 9%)	26	12% (SE 6%)	0.467
	4	6	‐	15	13% (SE 9%)	21	10% (SE 6%)	0.193
	Total	31	19% (SE 7%)	63	29% (SE 6%)	54	35% (SE 6%)	0.085
	*P*	0.009		0.007		<0.001		

Visceral fat area								
		Normal		High		Total		*P*
SIG	0	6	‐	5	‐	11	64% (SE 15%)	0.091
	1	5	‐	12	42% (SE 14%)	17	41% (SE 12%)	0.347
	2	5	‐	13	31% (SE 13%)	18	22% (SE 10%)	0.068
	3	7	‐	19	16% (SE 8%)	26	12% (SE 6%)	0.494
	4	5	‐	13	15% (SE 10%)	21	10% (SE 6%)	0.028
	Total	31	15% (SE 7%)	62	29% (SE 6%)	54	35% (SE 6%)	0.036
	*P*	<0.001		0.019		<0.001		

Skeletal muscle index								
		Normal		Low		Total		*P*
SIG	0	5	‐	6	‐	11	64% (SE 15%)	0.034
	1	8	‐	9	‐	17	41% (SE 12%)	0.323
	2	4	‐	14	14% (SE 9%)	18	22% (SE 10%)	0.200
	3	9	‐	17	6% (SE 6%)	26	12% (SE 6%)	0.029
	4	4	‐	17	12% (SE 8%)	21	10% (SE 6%)	0.540
	Total	30	43% (SE 9%)	63	16% (SE 5%)	54	35% (SE 6%)	<0.001
	*P*	0.003		0.139		<0.001		

Skeletal muscle density								
		Normal		Low		Total		*P*
SIG	0	4	‐	7	‐	11	64% (SE 15%)	0.141
	1	9	‐	8	‐	17	41% (SE 12%)	0.594
	2	7	‐	11	36% (SE 15%)	18	22% (SE 10%)	0.026
	3	7	‐	19	11% (SE 7%)	26	12% (SE 6%)	0.949
	4	4	‐	17	12% (SE 8%)	21	10% (SE 6%)	0.944
	Total	31	32% (SE 8%)	62	21% (SE 5%)	54	35% (SE 6%)	0.366
	*P*	0.004		0.019		<0.001		

#### SFI

Within the overall cohort of patients, SFI did not have a significant effect on survival (3‐yr OS 19% vs 29%, *P* = 0.085). When patients were subgrouped by SIG, an independent effect of SFI on survival was seen in the SIG 1 subgroup (*P* = 0.011). SIG was associated with worse survival independent of SFI in both the normal SFI and high SFI subgroups (*P* = 0.009 and *P* = 0.007, respectively).

#### VFA

Within the overall cohort of patients, high VFA was associated with a significantly better survival (3‐yr OS 29% vs 15%, *P* = 0.036). When patients were subgrouped by SIG, an independent effect of VFA on survival was seen in the SIG 4 subgroup (*P* = 0.028). SIG was associated with worse survival independent of VFA in both the normal VFA and high VFA subgroups (*P* < 0.001 and *P* = 0.019, respectively).

#### SMI

Within the overall cohort of patients, low SMI was associated with worse survival (3 yr OS 43% vs 16%, *P* < 0.001). When patients were subgrouped by SIG an independent effect of SMI on survival was seen in the SIG 0 cohort (*P* = 0.034) and SIG 3 cohort (*P* = 0.029). SIG was associated with worse survival independent of SMI in the normal SMI subgroup (*P* = 0.003).

#### SMD

Within the overall cohort of patients, SMD did not have a significant effect on survival (3‐yr OS 325 vs 21%, *P* = 0.366). When patients were subgrouped by SIG, an independent effect of SMD on survival was only seen in the SIG 2 cohort (*P* = 0.026). SIG was associated with worse survival independent of SMD in both the normal SMD and low SMD groups (*P* = 0.004/0.019).

## Discussion

The results of the present study show that CT‐derived body composition changes are highly prevalent in colon cancer and are associated with both the SIR and TNM stage. Body composition, in particular a low visceral fat area and low skeletal muscle index, is independently associated with emergency presentation after adjustment for other common clinicopathological characteristics. Finally, both SIG and markers of CT‐derived body composition while being associated retain independent prognostic value for 3‐year overall survival after adjustment for tumour stage, both in patients undergoing curative surgery for TNM II–III colon cancer and in patients with TNM IV colon cancer.

The high proportion of abnormal CT‐derived anthropometric measurements in patients with colon cancer is in keeping with previous literature.^17^, ^18^ A recent review^2^ reported a clear association between the SIR and markers of body composition and the present results confirm this. However, McSorley et al.^19^ previously reported no association between CT‐derived anthropometric measurements and TNM stage after adjustment for the SIR (as measured by mGPS). Within the present study, findings varied for different anthropometric measurements and subgroups of SIG; however, an association with TNM stage was observed, independent of the SIR. Although the present study included patients with TNM Stage IV disease, McSorley et al. did not include patients with metastatic disease. The present results show a stepwise change between metastatic and non‐metastatic colon cancer, particularly within CT‐derived fat indices (SFI/VFA) potentially explaining this discrepancy in findings. Furthermore, McSorley et al. grouped patients by TNM stage into node‐negative (TNM I–II) and node‐positive (TNM III) disease. The present results show that within non‐metastatic patients, TNM I and TNM II disease represented each end of the body composition spectrum with TNM Stage III disease representing intermediate values. This may suggest that the tumour component as opposed to nodal component of TNM staging is more closely correlated with measurements of CT‐derived body composition.

After adjustment for TNM stage, SIG readily stratified overall survival in this cohort. Whereas some of the prognostic value of CT‐derived body composition is less apparent after adjustment for SIG and TNM stage, body composition, in particular SMD, remained independently significant. SMD has been less widely reported than SMI but does have clear prognostic implications both in the present study and in previous studies.^20^, ^21^ Often termed myosteatosis, literally abnormal infiltration of fat into muscle tissue,^22^ the pathophysiology of low skeletal muscle density remains poorly understood and requires further investigation.

To our knowledge, the association between markers of CT‐derived body composition and mode of presentation of colon cancer has not been previously studied. It is widely recognized that emergency presentations of colon cancer are associated with worse long‐term outcomes than elective presentations^23^, ^24^, ^25^ even after adjustment for other factors including TNM stage. The present findings show the independent association of VFA and SMI with mode of presentation, and this may in part explain the discrepancy in outcomes between elective and emergency presentations.

Limitations of the present study include the retrospective nature of this study and the need to exclude patients with missing data, in particular BMI or pre‐operative laboratory results. TNM Stage I colon cancer carries an excellent prognosis, and there were very few TNM Stage I patients with SIG > 2. The present study was underpowered to assess this group. Furthermore, within TNM Stage IV colon cancer, patient numbers were low as it was not possible to stratify CT‐derived body composition due to lack of BMI data, and as a result, this group could not be subclassified into modality of treatment received. It is clear that abnormal markers of CT‐derived body composition are highly prevalent within colon cancer. The CT‐derived body composition of this cohort prior to developing colon cancer is unknown—little is known about CT‐derived body composition within the healthy population, and longitudinal studies of this nature comparing body composition in the healthy population compared with those with cancer would be of interest. Within this study, patients with TNM Stage I–III colon cancer were only included if they were undergoing curative surgery. Patients with TNM Stage IV disease were included regardless of what treatment they were undergoing, and this discrepancy may introduce the possibility of bias between these groups. Due to data availability, it was not possible to compare body composition between groups either undergoing or deemed unfit for potentially curative surgery; however, this would be of interest in future studies.

Low lean muscle mass is clearly associated with adverse outcomes in colon cancer. Resulting from a likely combination of obesity, low physical activity and the SIR, these patients have poor oncological outcomes. Body composition and the systemic inflammatory response are therefore potential targets to manipulate with the aim of improving these outcomes. One such strategy is pre‐habilitation using structured exercise programmes in the intervening period between diagnosis and surgery.^26^ However, major obstacles exist in terms of the short window of opportunity from diagnosis to surgery and the high levels of patient engagement required. Furthermore, based on available evidence, benefits remain uncertain. Although some studies have demonstrated an improvement in pre‐operative functional capacity with exercise training,^27^, ^28^ the effect of pre‐habilitation on the SIR and short‐term/long‐term outcomes is unclear.^29^, ^30^, ^31^ Alternatively, pharmacological manipulation of the SIR may improve lean muscle mass and outcomes. These strategies are likely to be relevant both to the metastatic and non‐metastatic cohort.

In conclusion, abnormal body composition is prevalent within both TNM I–III and advanced (TNM IV) colon cancer and associated with the systemic inflammatory response, mode of presentation, TNM stage and adverse outcomes. Further research is required to determine whether interventions including structured exercise programmes or attenuation of the SIR has a demonstrable effect on CT‐derived body composition and subsequently oncological outcomes.

## Conflict of interest

Allan Golder, Ling Kwan Ernest Sin, Fatima Alani, Ala Alasadi, Ross Dolan, David Mansouri, Paul Horgan, Donald McMillan and Campbell Roxburgh declare that they have no conflicts of interest.

## Funding

Nil.
